# Texture analysis of acute myocardial infarction with CT: First experience study

**DOI:** 10.1371/journal.pone.0186876

**Published:** 2017-11-02

**Authors:** Ricarda Hinzpeter, Matthias W. Wagner, Moritz C. Wurnig, Burkhardt Seifert, Robert Manka, Hatem Alkadhi

**Affiliations:** 1 Institute of Diagnostic and Interventional Radiology, University Hospital Zurich, University of Zurich, Zurich, Switzerland; 2 Epidemiology, Biostatistics and Prevention Institute, University of Zurich, Zurich, Switzerland; 3 Department of Cardiology, University Heart Center Zurich, University of Zurich, Zurich, Switzerland; 4 Institute for Biomedical Engineering, University and ETH Zurich, Zurich, Switzerland; Universidad Francisco de Vitoria, SPAIN

## Abstract

**Objective:**

To investigate the feasibility and accuracy of texture analysis to distinguish through objective and quantitative image information between healthy and infarcted myocardium with computed tomography (CT).

**Materials and methods:**

Twenty patients (5 females; mean age 56±10years) with proven acute myocardial infarction (MI) and 20 patients (8 females; mean age 42±15years) with no cardiac abnormalities (hereafter termed *controls*) underwent contrast-enhanced cardiac CT. Short axis CT images of the left ventricle (LV) were reconstructed at the slice thicknesses 1mm, 2mm, and 5mm. Two independent, blinded readers segmented the LV in controls and patients. Texture analysis was performed yielding first-level features based on the histogram (variance, skewness, kurtosis, entropy), second-level features based on the gray-level co-occurrence matrix (GLCM) (contrast, correlation, energy and homogeneity), and third-level features based on the gray-level run-length matrix (GLRLM).

**Results:**

Inter-and intrareader agreement was good to excellent for all histogram (intraclass correlation coefficient (ICC):0.70–0.93) and for all GLCM features (ICC:0.66–0.99), and was variable for the GLRLM features (ICC:-0.12–0.99). Univariate analysis showed significant differences between patients and controls for 2/4 histogram features, 3/4 GLCM and for 6/11 GLRLM features and all assessed slice thicknesses (all,p<0.05). In a multivariate logistic regression model, the single best variable from each level, determined by ROC analysis, was included stepwise. The best model included kurtosis (OR 0.08, 95%CI:0.01–0.65,*P* = 0.018) and short run high gray-level emphasis (SRHGE, OR 0.97, 95%CI:0.94–0.99,*P* = 0.007), with an area-under-the-curve (AUC) of 0.90 (95%CI:0.80–0.99). The best results for kurtosis and SRHGE (AUC = 0.78) were obtained at a 5mm slice thickness. A cut-off value of 14.4 for kurtosis+0.013*SRHGE predicted acute MI with a sensitivity of 95% (specificity 55%).

**Conclusion:**

Our study illustrates the feasibility of texture analysis for distinguishing healthy from acutely infarcted myocardium with cardiac CT using objective, quantitative features, with most reproducible and accurate results at a short axis slice thickness of 5mm.

## Introduction

Cardiac computed tomography (CT) has developed into a routinely used imaging tool for detecting and ruling-out coronary artery stenosis [1, 2], providing also incremental prognostic information to the patients [3]. In addition to the coronary arteries, cardiac CT has been also tested for evaluating the myocardium. Initial studies performed already four decades ago tested the general ability of CT for detecting myocardial infarction in excised hearts of mongrel dogs [4, 5]. In 1978, Gray et al. [6] were one of the first groups to evaluate the potential of CT to identify, accurately localize and quantitate experimental acute myocardial infarction (MI) in isolated canine hearts. However, in spite of these early promising results combined with the tremendous developments of the technology in the past years, CT still has shortcomings when evaluating the myocardium. This is mainly due to the inherently low contrast resolution of CT compared to the reference standard modality magnetic resonance (MR) imaging [7]. To compensate for this disadvantage, a higher contrast-to-noise ratio (CNR) can be reached by administering larger volumes of contrast media [8]. Another option is the application of dual-energy CT allowing for a direct separation of iodine from myocardial tissue [9]. This technique has been suggested to allow for a better determination of myocardial regions with ischemia or infarction from those with a normal perfusion [10].

Texture analysis (TA) refers to an objective and quantitative set of metrics calculated for quantifying the textural patterns of images. TA converts radiological images into a multi-dimensional mineable feature space using automatically extracted data characterization algorithms [11]. Such TA features might detect distinct quantifiable phenotypic differences of tissues which cannot be assessed through a qualitative, visual evaluation of radiological images alone. The texture features can be increasingly used for computer-aided pattern recognition and classification techniques [12]. So far, TA has shown value in the differentiation of fat-poor angiomyolipoma from renal cell carcinoma on unenhanced CT examinations [13] and for differentiating benign and malignant mediastinal lymph nodes in lung cancer [14]. Another study showed that CT-based whole tumor TA features were related to 5-year overall survival in patients with colorectal cancer [15]. In a recent preliminary study, Thornhill and colleagues evaluated the ability of TA to distinguish between the hearts of healthy volunteers and patients with hypertrophic cardiomyopathy and found significant differences of certain features between groups [16].

Various parameters have been reported to influence the results from TA, including the application of contrast media, different contrast media phases and image post-processing parameters such as the reconstructed slice thickness [17] and spatial resolution [12]. Ganeshan et al. [15] showed significant differences of TA features between non-enhanced and contrast-enhanced CT images of lung cancer. Other authors showed the influence of CT slice thicknesses on certain TA parameters for assessing the microarchitecture of bones [18]. In distinction, MR imaging of the brain showed only minor differences in TA features between various slice thicknesses [19].

To our knowledge, no study so far investigated the ability of TA in CT studies of the heart nor evaluated the influence of slice thickness on the derived TA features. Objective and quantitative metrics characterizing images, however, appear desirable for a potential improvement of the pure visual assessment of the myocardium with CT. Being designed as a proof of concept study, we choose patients with acute MI as the pathological model for demonstrating the feasibility of TA. Thus, the purpose of our study was to evaluate the ability of TA to distinguish between healthy and acutely infarcted myocardium in CT based on objective and quantitative image information.

## Materials and methods

### Patient population

A database research between January 2014 and December 2015 was performed for identifying 20 patients (15 males, 5 females; mean age 56±10 years; range: 38–78 years) who underwent clinically indicated electrocardiography (ECG)-gated, contrast-enhanced cardiac CT and who were diagnosed with acute MI. The diagnosis of acute MI was based on the results from laboratory biomarkers, ECG, and other imaging tests including catheter coronary angiography and MR imaging during the time of index hospitalization. The segments with myocardial infarction were determined using the 17-segment model of the American Heart Association (AHA), and infarcts were subdivided into transmural MI and non-transmural MI (**Table 1**). The indication for CT in these emergency department patients was acute chest pain and initially inconclusive ECG and biomarkers.

**Table 1:** Demographic data of the patients with acute MI subdivided by gender.

	Patients with acute MI		
	male	female	p-value
	n = 15 (75%)	n = 5 (25%)	
Age (years) (mean ± SD)	55 ± 10	61 ± 12	0.35
Heart rate during CT	80 ± 23 bpm	83 ± 33 bpm	1.0
Sensitive Troponin T positive	13/15 (87%)	5/5 (100%)	0.67
ECG positive for STEMI	6/20 (40%)	2/5 (40%)	0.57
Affected Vessels			
LAD	10	4	
CX	10	1	
RCA	5	1	
Number of affected vessels (mean)	2	1.4	0.19
Number of affected myocardial segments			0.67
2 segments	8/15 (53%)	2/5 (40%)	
3 segments	1/15 (7%)	1/5 (20%)	
4 segments	5/15 (33%)	1/5 (20%)	
5 segments	0/15 (0%)	1/5 (20%)	
7 segments	1/20 (7%)	0/5 (0%)	
Transmurality	8/15 (53%)	3/5 (60%)	0.87

CT: computed tomography, ECG: electrocardiography, STEMI: ST-segment elevation myocardial infarction, LAD: left anterior descending, CX: circumflex artery, RCA: right coronary artery

As the control population, additional 20 patients (12 males, 8 females; mean age 42±15 years; range: 19–55 years) who underwent cardiac CT in the same time period with the same CT scanner and same protocol were included. The indication of CT in the control group was atypical chest pain with a low pre-test probability of coronary artery disease. In these patients, coronary CT angiography showed no plaques and no stenoses. All patients from the control group underwent echocardiography within 2 days showing no abnormality, and had no history of cardiac disease or surgery/intervention. Reasons for atypical chest pain in the controls were gastroesophageal reflux in 4/20 (20%), cholecystolithiasis in 1/20 (5%), and musculoskeletal pain in 15/20 patients (75%).

This study had institutional review board and local ethics committee approval. All investigations were conducted according to the Declaration of Helsinki. Written informed consent requirement was waived by the local ethics committee because of the retrospective nature of the study.

### CT data acquisition and image post-processing

CT examinations were performed on a second generation dual-source CT scanner (SOMATOM Flash, Siemens Healthineers, Forchheim, Germany). Acquisition parameters were as follows: detector collimation 2x64 mm, slice acquisition 2x128 mm using the z-flying focal spot, gantry rotation time 280 ms, pitch 0.2–0.5, tube voltage 100 kVp, quality reference tube current-time product 250 mAs per rotation using automated exposure control (CareDose), effective tube current-time product 152–399 mAs per rotation. Data acquisition was prospectively synchronized to the ECG in the step-and-shoot mode. The contrast media protocol was as follows: first, 80–100 ml nonionic iodinated contrast media (iopromidum, Ultravist 370, 370mg/ml, Bayer, Leverkusen, Germany) was injected in an antecubital vein with a flow rate of 5–6 ml/sec, depending on the patients’ body mass index, followed by the same volume consisting of 20% contrast media and 80% saline solution. No beta blockers were administered; all patients received sublingual nitroglycerine (2 strokes sublingual, Isosorbiddinitrat, Isoket Spray, 25mg/ml, UCB-Pharma, Brussels, Belgium) prior to CT.

**Fig 1:**
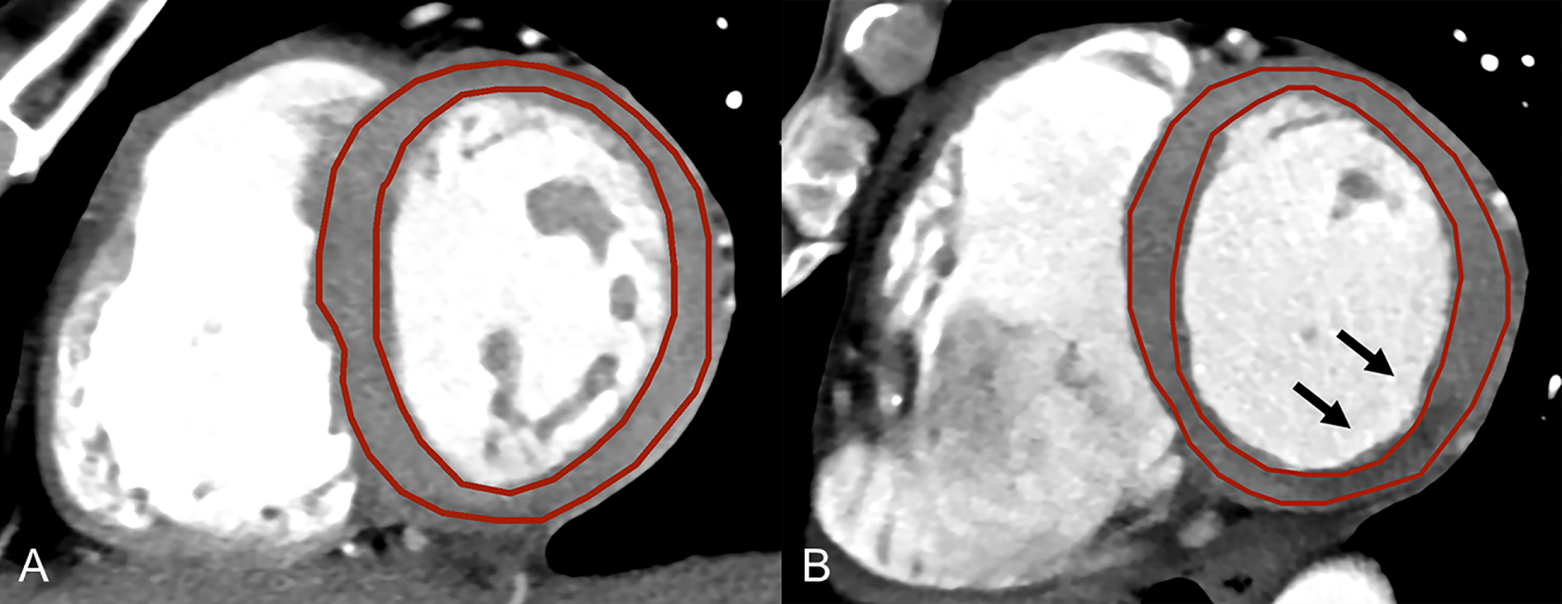
Reformatted short axis images of the left ventricle. Reformatted short axis images of the left ventricle at a slice thickness of 5 mm in a control (**A**) and in a patient with acute myocardial infarction (**B**) illustrating the free-hand regions-of-interest for texture analysis. Note the septal hypodensity indicating myocardial infarction (arrows).

Axial CT images were reconstructed with a slice thickness of 0.75 mm (increment 0.4 mm) using a medium smooth convolution kernel with sinogram-affirmed iterative reconstruction (I30f) at a strength level of 3, with a field-of-view of 200x200 mm^2^ and an image matrix of 512x512. From these axial image data, short axis images of the left ventricle (LV) were reformatted at three different slice thicknesses (1 mm, 2 mm, and 5 mm) using commercially available post-processing software (CT Cardiac Function, syngo.via, Siemens). In this feasibility study, we choose in patients with MI the respective short axis image showing the largest extent of infarction. In controls, short axis images in mid-ventricular myocardium were chosen. In order to standardize reading and subsequent TA, images were standardized to a window level and width of 300 Hounsfield Units (HU) and 30 HU, respectively. These reformatted images were anonymized and stored in Digital Imaging and Communications in Medicine (DICOM) file format by using the same software. Then, regions-of-interest (ROIs) were drawn freehand by two independent and blinded readers (with 2 and 3 years of experience in cardiac radiology, respectively) including the LV myocardium in both controls and in patients with MI. Care was taken to avoid including LV blood or epicardial fat in the ROI (**Fig 1**). The ROI was delineated on images with 1 mm slice thickness and were copied to the other slices with a thickness of 2 mm and 5 mm, so as to avoid confounding of the TA results through variability in the definition of the ROI. To determine the intrareader agreement, reader 1 repeated delineation of the ROIs 24h after the initial definition using the same data set.

### Texture analysis

**Fig 2:**
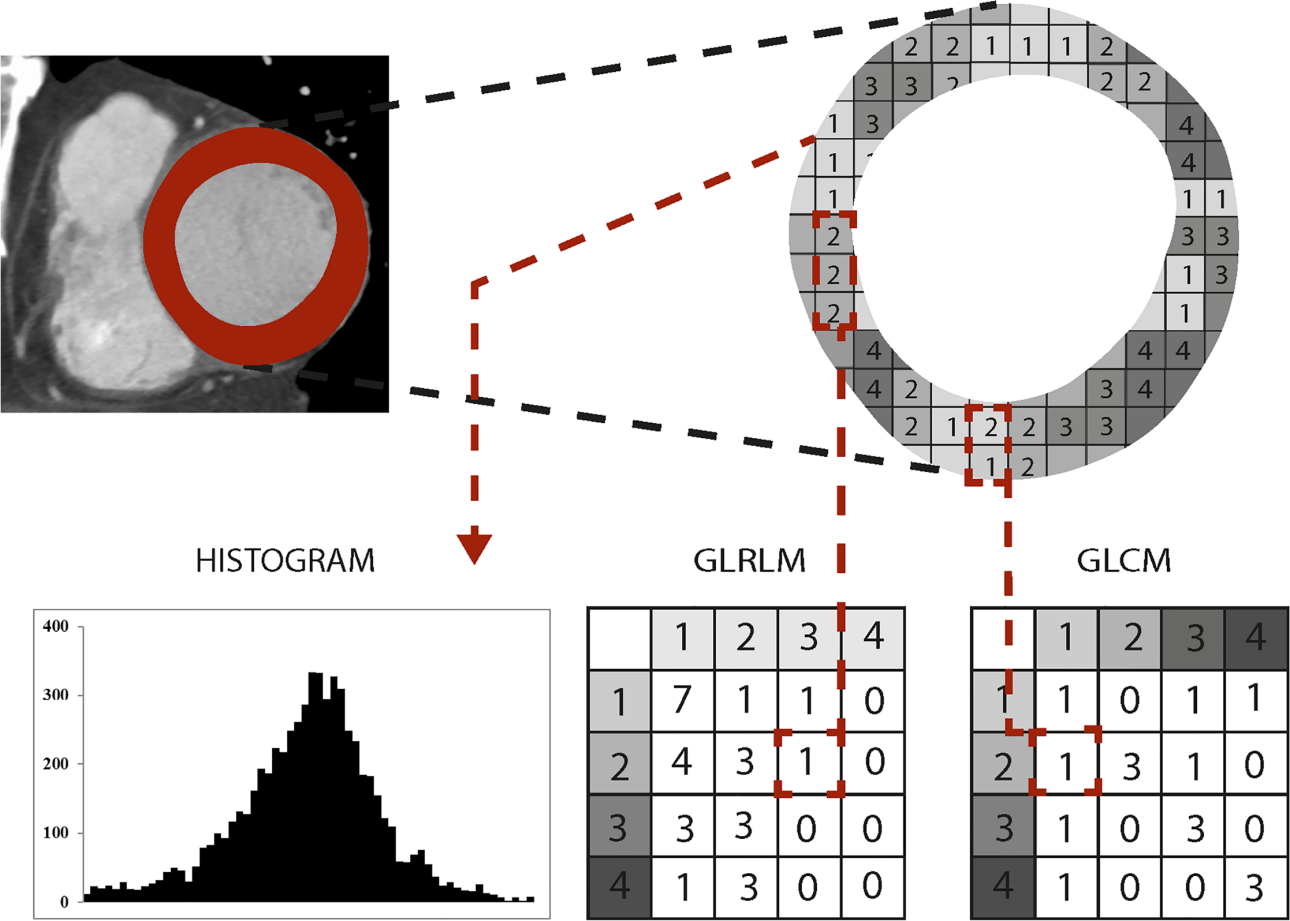
Principles of generating texture analysis features. Principles of generating the histogram and the variables gray-level run-length matrix (GLRLM) and gray-level co-occurrence matrix (GLCM) from a given ROI. To construct the GLCM each pixel of the ROI is once compared with a pixel in a given distance and direction (0°, 45°, 90° or 135°). For each value pair the GLCM is increased by 1 in the respective column and row (in the given example a distance of 1 and direction of 90° were used; one 2–1 pair was found and 1 was added to the matrix accordingly). Runs of the same grey-level in a given direction (0°, 45°, 90° or 135°) are assessed to construct the GLRLM and used as the x-axis in the matrix, whereas the y-axis contains the assessed grey-levels. The example shows one run of three 2’s and a 1 is added to the matrix accordingly (a direction of 90° was used).

**Fig 3:**
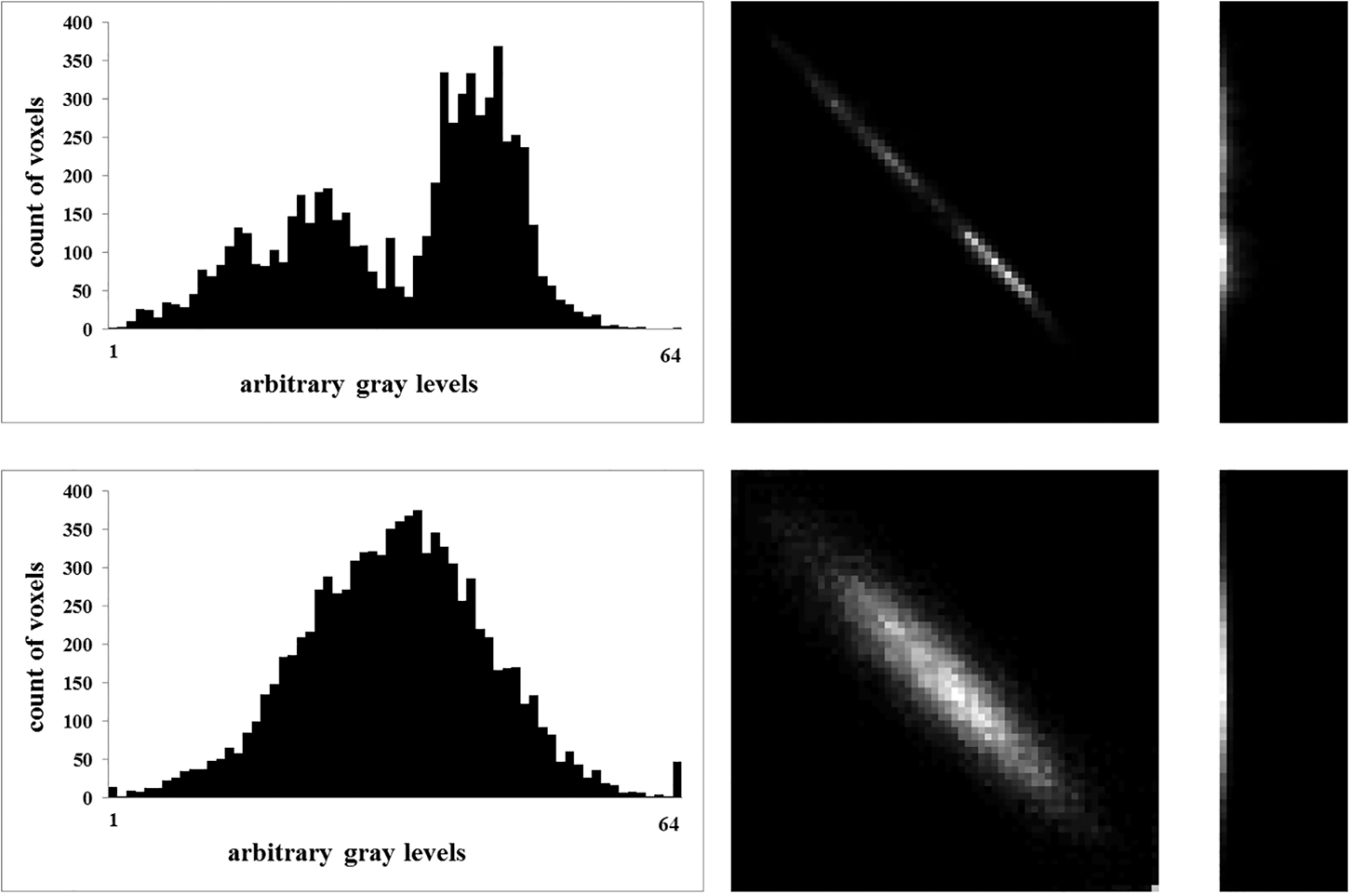
Differences of texture analysis features in controls and patients with acute MI. First- (histogram, **left column**), second- (GLCM, **middle column**), and third-level (GLRLM, **right column**) texture analysis features in a patient with acute myocardial infarction (**upper row**) and in a control (**lower row**). Note the additional peak at lower gray levels in patients with acute myocardial infarction indicating the proportion of voxels with a lower density and the divergent distribution of voxels in GLCM and GLRLM between controls and patients.

Texture analysis was implemented using institutional [20] computer scripts written in Matlab (The MathWorks, Natick, MA) based on prior algorithms developed by Wei [21] and later applied by Vallieres et al. [22]. The first step of TA was normalization: ROI contents were normalized between μ ± 3σ (μ = grey level mean, σ = grey level standard deviation). Then, grey levels between ± 3σ were decimated to 64 grey levels. This method has been previously used for minimizing intra- and interscanner effects [22]. After this normalization and requantization to a 6-bit image, the following features were computed: Histogram (i.e. first-level) features of all gray-levels in the ROI were calculated including the variables variance, skewness, kurtosis and entropy. As previously described by Haralick et al. [23], the gray-level co-occurrence matrix (GLCM) was created based on the gray-level distribution within the ROI. By using the GLCM, the second-level features contrast, correlation, energy and homogeneity were obtained. Finally, the gray-level run-length matrix (GLRLM) was created as introduced by Galloway [24]. Using the GLRLM, the third-level features short run emphasis (SRE), long run emphasis (LRE), gray-level nonuniformity (GLN), run-length nonuniformity (RLN), run percentage (RP), low gray-level run emphasis (LGRE), high gray-level run emphasis (HGRE), short run low gray-level emphasis (SRLGE), short run high gray-level emphasis (SRHGE), long run low gray-level emphasis (LRLGE), and long run high gray-level emphasis (LRHGE) were derived as described in several previous publications [24–26]. The general principle for generating the second- and third-level matrices GLCM and GLRLM are illustrated in **Fig 2**, the corresponding images are shown in **Fig 3**. GLCM and GLRLM features were computed in four directions differing from each other in a 45 degree angle. The four resulting matrices were averaged to avoid direction dependency and to acquire a global view of the texture information [27]. Texture features were determined for further analyses. Once the ROI placement was finished, calculation of the texture features was performed by the software (installed on a standard personal computer) within a few seconds, not requiring large computing capacity.

### Statistical analysis and feature reduction

The intra- and interreader agreement for all TA features and different slice thicknesses was assessed using intra-class correlation coefficients (ICC). An ICC of 0.75–1 indicated excellent agreement, 0.60–0.74 good agreement, 0.40–0.59 fair agreement, and below 0.4 poor agreement [28]. Differences between gender regarding demographic parameters were calculated using the non-parametric Mann-Whitney-U-test. First, we used again the non-parametric Mann-Whitney U-test (because of the small sample size) for detecting differences in TA features between controls and patients with acute MI. Then, we sought to identify the most accurate feature from the first and second level using receiver operating characteristics (ROC) analysis for each TA feature and each slice thickness. For the third level features, we reduced the number of features using a Pearson correlation for excluding those features showing similar values, hereby reducing redundancy in the data. ROC analysis was made on the remaining feature set for each slice thickness to extract the most accurate third level feature. Then, tests of equality of ROC curves were used to determine the three most accurate TA features (one for each level).

A stepwise forward logistic regression analysis with acute MI as dependent variable and the three most accurate features was then performed (see results section below for reasons why 5 mm short axis reformations were used). Goodness-of-fit of the logistic regression model was evaluated using the Hosmer-Lemeshow test. ROC analysis for the final model and the single most accurate TA features from each level were constructed and the area-under-the-curve (AUC) including the 95% confidence intervals (CI) were computed. Cut-off values for the most accurate TA features for diagnosing acute MI were calculated. Statistical analyses were performed using IBM SPSS Statistics (Version 22.0 Armonk, NY: IBM Corp) and STATA (StataCorp LP, version 13.1, TX, USA). A two-tailed p-value below 0.05 was considered to infer statistical significance.

## Results

### Patient population

Twenty patients (15 males, 5 females; mean age 56±10 years; range: 38–78 years) who underwent clinically indicated ECG-gated, contrast-enhanced cardiac CT and who were diagnosed with acute MI were identified. There were no significant differences between genders regarding age (p = 0.35), heart rate during CT (p = 1.0), positive sensitive troponin T (p = 0.67), STEMI on ECG (p = 0.57), number of affected vessels (p = 0.2), number of affected myocardial segments (p = 0.67), and the presence or absence of transmural MI (P = 0.87) (**Table 1**).

### Inter- and intrareader agreement

All results regarding inter- and intrareader agreement can be found in **Table 2**. For all slice thicknesses, the agreement was good to excellent for all first-level (interreader: ICC = 0.70–0.93, intrareader: ICC = 0.70–0.91) and all second-level features (interreader: ICC = 0.66–0.98, intrareader: ICC = 0.66–0.98). For the third-level features inter- and intrareader agreement ranged between poor and excellent (interreader: ICC = -0.12–0.99, intrareader: ICC = 0.30–0.99).

**Table 2:** Intraclass correlation coefficients indicating the inter- and intrareader variability of all texture analysis features at each slice thickness.

		Interreader Variability	Intrareader Variability	Interreader Variability	Intrareader Variability	Interreader Variability	Intrareader Variability
		1mm		2mm		5mm	
	**variance**	**0.888**	**0.837**	**0.905**	**0.846**	**0.695**	**0.703**
**First-level**	**skewness**	**0.873**	**0.818**	**0.863**	**0.819**	**0.833**	**0.844**
**features**	**kurtosis**	**0.934**	**0.896**	**0.929**	**0.905**	**0.908**	**0.913**
	**entropy**	**0.854**	**0.761**	**0.826**	**0.730**	**0.740**	**0.787**
	**contrast**	**0.966**	**0.968**	**0.950**	**0.960**	**0.919**	**0.950**
**Second-level**	**correlation**	**0.971**	**0.968**	**0.962**	**0.967**	**0.933**	**0.951**
**features**	**energy**	**0.796**	**0.796**	**0.661**	**0.661**	**0.818**	**0.818**
	**homogeneity**	**0.988**	**0.979**	**0.983**	**0.973**	**0.973**	**0.971**
	**SRE**	**0.982**	**0.968**	**0.982**	**0.969**	**0.976**	**0.963**
	**LRE**	**0.990**	**0.976**	**0.985**	**0.970**	**0.980**	**0.964**
	**GLN**	**0.948**	**0.971**	**0.944**	**0.969**	**0.933**	**0.966**
	**RLN**	**0.966**	**0.986**	**0.966**	**0.987**	**0.971**	**0.990**
**Third-level**	**RP**	**0.988**	**0.978**	**0.983**	**0.970**	**0.977**	**0.969**
**features**	**LGRE**	**0.615**	0.455	**0.649**	**0.649**	**0.615**	0.565
	**HGRE**	**0.649**	**0.762**	**0.748**	**0.798**	**0.787**	**0.749**
	**SRLGE**	-0.157	0.451	0.333	0.333	-0.115	0.253
	**SRHGE**	**0.977**	**0.972**	**0.975**	**0.967**	**0.969**	**0.951**
	**LRLGE**	**0.616**	0.300	**0.664**	0.342	**0.647**	**0.632**
	**LRHGE**	**0.991**	**0.981**	**0.986**	**0.972**	**0.981**	**0.964**

SRE: short run emphasis, LRE: long run emphasis, GLN: gray-level nonuniformity, RLN: run-length nonuniformity, RP: run percentage, LGRE: low gray-level run emphasis, HGRE: high gray-level run emphasis, SRLGE: short run low gray-level emphasis, SRHGE: short run high gray-level emphasis, LRLGE: long run low gray-level emphasis, LRHGE: long run high gray-level emphasis. All variables showing a good (>0.60) or higher agreement are indicated in **bold**.

### TA features: 1mm slice thickness

Comparison of first-level features between controls and patients with acute MI showed significant differences for the variance (*P =* 0.018) and kurtosis (*P =* 0.014). Second level features showed significant differences for contrast (*P =* 0.004), correlation (*P =* 0.006) and homogeneity (*P =* 0.005). Third level features showed significant differences for SRE (*P =* 0.004), LRE (*P =* 0.009), RP (*P =* 0.007), HGRE (*P =* 0.049), SRHGE (*P =* 0.001) and LRHGE (*P =* 0.003). All other variables showed no significant differences between groups (**Table 3**).

**Table 3:** Comparison of texture analysis features between controls and patients with acute MI for different slice thicknesses. Values present the median and the interquartile range in parentheses.

		Control	MI		Control	MI		Control	MI	
		1mm		p-value	2mm		p-value	5mm		p-value
	**Variance**	112.02 (2.83)	113.13 (10.86)	**0.018**	112.27 (2.74)	113.29 (18.10)	**0.017**	112.24 (3.65)	114.26 (23.19)	**0.008**
**First-level**	**Skewness**	-0.04 (0.22)	-0.15 (0.47)	0.28	-0.06 (0.32)	-0.15 (0.48)	0.36	-0.09 (0.46)	-0.16 (0.49)	0.43
**features**	**Kurtosis**	0.15 (0.31)	-0.11 (0.63)	**0.014**	0.13 (0.46)	-0.19 (0.83)	**0.014**	0.25 (0.61)	-0.29 (1.04)	**0.002**
	**Entropy**	5.36 (0.06)	5.34 (0.10)	0.37	5.34 (0.06)	5.31 (0.10)	0.45	5.31 (0.09)	5.28 (0.12)	0.53
	**Contrast**	20.92 (18.65)	13.38 (11.91)	**0.004**	17.73 (14.09)	9.25 (9.69)	**0.001**	12.80 (9.90)	6.04 (5.75)	**<0.001**
**Second-level**	**Correlation**	0.91 (0.08)	0.94 (0.05)	**0.006**	0.92 (0.06)	0.96 (0.04)	**0.001**	0.94 (0.04)	0.97 (0.03)	**<0.001**
**features**	**Energy**	0.00 (0.00)	0.00 (0.00)	0.80	0.00 (0.00)	0.00 (0.00)	0.80	0.00 (0.00)	0.00 (0.01)	0.43
	**Homogeneity**	0.35 (0.08)	0.39 (0.10)	**0.005**	0.37 (0.07)	0.44 (0.11)	**0.003**	0.40 (0.09)	0.49 (0.11)	**0.001**
	**SRE**	0.93 (0.02)	0.91 (0.04)	**0.004**	0.92 (0.02)	0.89 (0.04)	**0.004**	0.91 (0.04)	0.86 (0.05)	**0.001**
	**LRE**	1.36 (0.18)	1.52 (0.35)	**0.009**	1.42 (0.21)	1.66 (0.43)	**0.005**	1.58 (0.38)	1.93 (0.59)	**0.006**
	**GLN**	312.12 (137.98)	367.54 (253.27)	0.55	312.79 (152.33)	359.45 (250.55)	0.57	316.38 (179.85)	349.67 (239.45)	0.64
	**RLN**	9108.79 (5347.96)	9883.15 (7812.59)	0.62	8979.50 (5159.15)	9313.33 (7303.64)	0.70	8115.29 (4695.15)	8203.88 (6609.40)	0.90
**Third-level**	**RP**	0.90 (0.04)	0.88 (0.07)	**0.007**	0.89 (0.04)	0.85 (0.07)	**0.004**	0.87 (0.06)	0.81 (0.08)	**0.003**
**features**	**LGRE**	0.00 (0.01)	0.00 (0.00)	0.43	0.00 (0.01)	0.00 (0.00)	0.18	0.00 (0.01)	0.00 (0.00)	0.30
	**HGRE**	1165.85 (7.06)	1162.88 (9.29)	**0.049**	1166.79 (9.59)	1160.54 (10.13)	**0.013**	1167.71 (7.82)	1162.17 (13.86)	**0.023**
	**SRLGE**	0.00 (0.00)	0.00 (0.00)	0.60	0.00 (0.00)	0.00 (0.00)	0.60	0.00 (0.00)	0.00 (0.00)	0.80
	**SRHGE**	1086.54 (23.57)	1047.94 (47.51)	**0.001**	1069.91 (25.43)	1025.72 (54.08)	**0.001**	1057.55 (42.76)	994.60 (69.23)	**<0.001**
	**LRLGE**	0.01 (0.01)	0.01 (0.01)	0.30	0.02 (0.02)	0.01 (0.02)	0.19	0.01 (0.05)	0.02 (0.02)	0.24
	**LRHGE**	1590.04 (175.91)	1846.52 (418.14)	**0.003**	1641.41 (241.11)	2007.78 (478.56)	**0.002**	1822.58 (409.40)	2371.89 (715.18)	**0.003**

SRE: short run emphasis, LRE: long run emphasis, GLN: gray-level nonuniformity, RLN: run-length nonuniformity, RP: run percentage, LGRE: low gray-level run emphasis, HGRE: high gray-level run emphasis, SRLGE: short run low gray-level emphasis, SRHGE: short run high gray-level emphasis, LRLGE: long run low gray-level emphasis, LRHGE: long run high gray-level emphasis. Significant p-values are indicated in **bold**.

### TA features: 2mm slice thickness

Comparison of first-level features between controls and patients with acute MI showed significant differences for the variance (*P =* 0.008) and kurtosis (*P =* 0.002). Second level features showed significant differences for contrast (*P<*0.001), correlation (*P<*0.001) and homogeneity (*P =* 0.001). Third level features showed significant differences for SRE (*P =* 0.001), LRE (*P =* 0.006), RP (*P =* 0.003), HGRE (*P =* 0.023), SRHGE (*P<*0.001) and LRHGE (*P =* 0.003). All other variables showed no significant differences between groups (**Table 3**).

### TA features: 5mm slice thickness

Comparison of first-level features between controls and patients with acute MI showed significant differences for variance (*P =* 0.017) and kurtosis (*P =* 0.014). Second level features showed significant differences for contrast (*P =* 0.001), correlation (*P =* 0.001) and homogeneity (*P =* 0.003). Third level features showed significant differences for SRE (*P =* 0.004), LRE (*P =* 0.005), RP (*P =* 0.004), HGRE (*P =* 0.013), SRHGE (*P =* 0.001) and LRHGE (*P =* 0.002). All other variables showed no significant differences between groups (**Table 3**).

### Selection of the most accurate TA features

**Fig 4:**
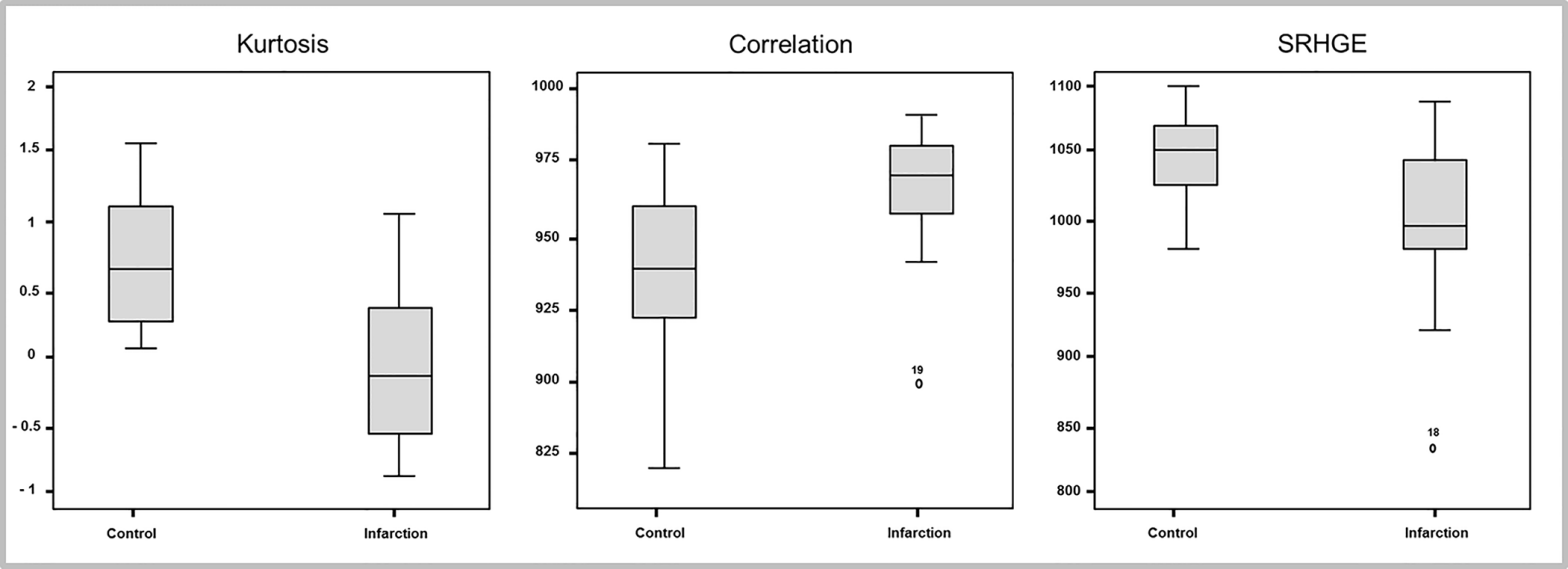
Boxplots of the three most distinguishing texture features. Boxplots showing the three most distinguishing TA features between patients with acute MI and controls on images reformatted with a 5 mm slice thickness.

The most accurate first-level feature based on ROC analysis was kurtosis (5mm slice thickness: AUC: 0.78, 95%CI 0.63–0.93, *P =* 0.002), the most accurate second-level feature was correlation (5mm slice thickness: AUC: 0.81, 95%CI 0.63–0.93, *P =* 0.002), and the most accurate third-level feature was SRHGE (5mm slice thickness: AUC: 0.82, 95%CI 0.68–0.95, *P =* 0.001) **(Fig 4)**.

### TA features across slice thicknesses

There were differences comparing the single best performing feature at each level across slice thicknesses (first-level: kurtosis, *P* = 0.20; second-level: correlation, *P* = 0.24; third-level: SRHGE, *P* = 0.89), however, without reaching statistical significance. The best results based on ROC analysis were obtained at a 5mm slice thickness for kurtosis (1mm: AUC = 0.73, 2mm: AUC = 0.73, 5mm: AUC = 0.78), correlation (1mm: AUC = 0.75, 2mm: AUC = 0.79, 5mm: AUC = 0.81) and SRHGE (1mm: AUC = 0.80, 2mm: AUC = 0.80, 5mm: AUC = 0.82). Thus, the quantitative variables from 5 mm short axis reformations were used for multivariate logistic regression analysis.

### Logistic regression analysis

The comparison of ROC curves indicated the variable kurtosis as the best first-level feature, correlation as the best second-level feature, and SRHGE as the best third-level feature. The stepwise logistic regression resulted in a model including kurtosis (OR 0.083, 95% CI 0.11–0.66; *P* = 0.018) and SRHGE (OR 0.97, 95%CI 0.94–0.99; *P =* 0.007). The Hosmer-Lemeshow test showed a good model fit (*P* = 0.94). The parameter correlation did not have an additional significant effect (*P* = 0.80).

**Fig 5:**
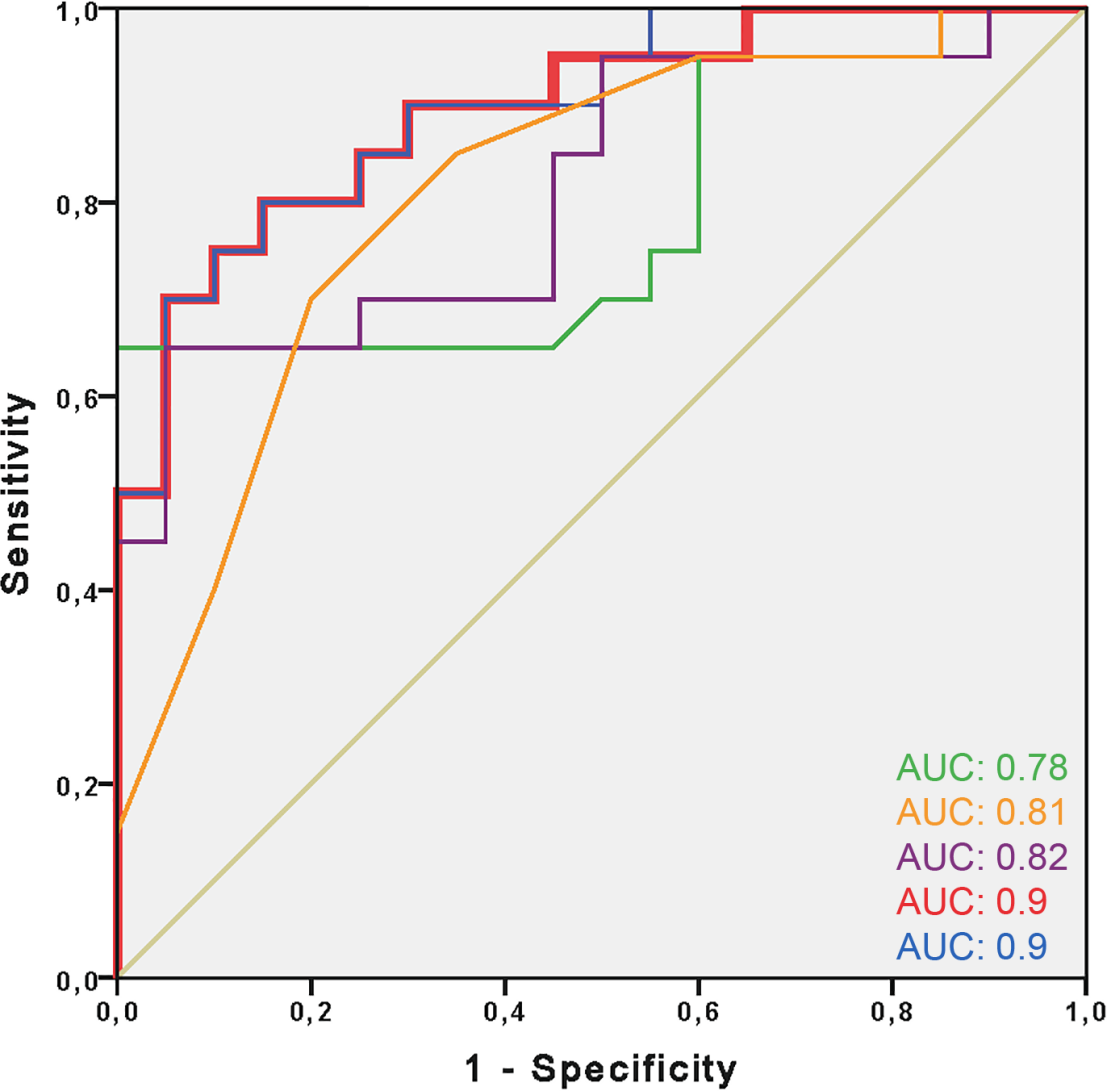
ROC analysis showing the best model for predicting acute MI. ROC analysis comparing the accuracy of the texture analysis features kurtosis (green; AUC: 0.78), correlation (orange; AUC: 0.81) and SRHGE (purple; AUC: 0.82) for predicting acute myocardial infarction. Combined analysis of kurtosis and SRHGE (red; mostly hidden behind the blue line, AUC: 0.9). Adding the parameter correlation to the first model added no benefit for the prediction of acute MI (blue; AUC: 0.9). Reference line in grey.

ROC analysis showed an AUC of 0.78 (95% CI 0.63–0.93) for kurtosis, 0.81 (95% CI 0.68–0.95) for correlation, and 0.82 (95% CI 0.68–0.95) for SRHGE. The logistic regression with kurtosis and SRHGE as predictors showed an AUC of 0.9 (95% CI 0.80–0.99). Adding the parameter correlation to the two other features did not improve the AUC (0.9; 95% CI 0.80–0.99) (**Fig 5**).

Based on ROC analysis, values higher or equal to a cut-off of 14.4, calculated as kurtosis + 0.013*SRHGE, is associated with a sensitivity of 95% (specificity 55%) for diagnosing acute MI.

## Discussion

TA describes an objective and quantitative means for quantifying the texture of images. Having the potential to demonstrate features that are not visible for the human eyes, there are also some image acquisition and data post-processing parameters that are known to influence the results from TA [12, 15, 17]. Our proof of concept study is the first–to our knowledge—to demonstrate the feasibility of TA in CT imaging of the heart, showing a good to excellent intra- and interreader agreement for all first and second-level features at all slice thicknesses. In distinction, the intra- and interreader agreement for some third-level features was poor, independent of the slice thickness. While the capabilities of the various features for distinguishing healthy from infarcted myocardium were not significantly related to slice thickness, the most accurate results were obtained at the highest slice thickness of 5 mm. Univariate analysis showed that the best quantitative parameters for distinguishing normal from infarcted myocardium were kurtosis (first-level), correlation (second-level), and SRHGE (third-level). Multivariate regression demonstrated that the combination of two of the features had a high accuracy for the diagnosis of acute MI. Importantly, these two identified features were highly reliable showing an excellent intra- and interreader agreement.

The TA methodology applied in this study demonstrated significant differences between healthy and infarcted myocardium for 2/4 first-level, 3/4 second-level and for 6/11 third-level features. The kurtosis, representing the first-level parameter with highest accuracy for differentiating between groups, showed a positive excess (median 0.25) in controls and a negative excess (median -0.29) in patients with acute MI. Kurtosis is a descriptor of the shape of a probability distribution and indicates whether a variable is heavy- or light-tailed relative to a normal distribution, the latter having a kurtosis of zero. Thus, the positive kurtosis of healthy myocardium indicates the distribution of voxels to be more peaked, whereas the negative kurtosis in infarcted myocardium indicates a flatter distribution which most probably is related to the second peak of voxel counts at lower density (**Fig 3**).

The parameter correlation, representing the second-level feature with highest accuracy for differentiating between groups, was higher (median 0.97) in patients with MI as compared to those with a normal myocardium (median 0.94). The parameter SRHGE, representing the third-level feature with highest accuracy for differentiating between groups, was lower (median 994.60) in patients with MI as compared to controls (median 1057.55). Unlike the features defined by Tamura et al. [29], who tried to define texture features corresponding to visual perception and to first-level features, which correspond to visually perceived distributions of gray levels, the meaning of second and third-level features is not readily apparent. However, similar to the observation of the second distinct peak at lower gray values in the histograms of patients with MI (**Fig 3**), both the GLCM and GLRLM showed two clusters in patients with MI instead of one in controls.

Previous studies indicated conflicting results regarding the influence of slice thickness on TA parameters. A study evaluating bone microarchitecture evaluated four different slice thicknesses from 1 to 8 mm showing significant changes of features [18]. While all GLRLM features decreased with increasing slice thickness, only one feature (long run emphasis (LRE)) increased. GLCM features either decreased, increased or showed no changes across slice thicknesses. In contrast, a study evaluating the effect of slice thickness on brain MR image TA parameters showed only minor differences of features across slice thicknesses [19]. The results of our study indicate no significant differences comparing all 19 TA features at all three levels and slice thicknesses. However, accuracy (and thus, the capability to differentiate between controls and patients with acute MI) was highest when using short axis reformations at a slice thickness of 5 mm. This is most probably related to the lower noise and higher contrast-to-noise ratio (CNR) in images reformatted with a higher slice thickness. This also corroborates with previous reports suggesting that changes in image acquisition parameters affecting image noise impinge especially finer texture scales [15].

Although TA-derived features are objective measures, it remains to be elucidated how stable these features are when different readers delineate slightly different ROIs. It can be assumed that histogram features are influenced to a lesser degree with larger ROIs and when gray levels are homogenously distributed throughout the ROI. In our study, first and second-level features showed a high intra- and interreader agreement, whereas third-level features were more variable within and between readers. We assume that the very sparse matrices and small deviations from third-level features are sensitive to variation and thus impact more on intra- and interreader agreement. As this observation was true for all assessed slice thicknesses, this variability seems to be inherent to the features and less influenced by noise and/or CNR.

A previous study investigating TA in cardiac CT indicated that the first level feature Energy is the most stable to differentiate between normal and scarred tissue [30]. In contrast, our study identified Kurtosis as the most accurate and distinguishing first level feature. Differences in CT scanner technology, scan protocol settings and image reconstruction, and a different age of MI might be the reason for these divergent findings.

TA in cardiac CT imaging provides a tool allowing for the investigation of differences between healthy subjects and patients with acute MI, currently not used in clinical routine but being potentially useful for objective, quantitative imaging. In our study, we explicitly did not segment only the areas of infarction but the entire LV myocardium with the rationale that the same is done also by automatic segmentation software tools of the left heart, such as for determining the LV myocardial mass and global systolic LV function. Thus, a possible clinical scenario would be that these automatic segmentations of the LV could be also used for texture analysis, which then indicates–based on the feature thresholds identified in this study–myocardial regions with possible infarction. Then, these regions would need to be re-analyzed by the radiologist for confirming the infarct and for searching the corresponding culprit coronary lesion. Theoretically, TA is feasible on any kind of CT image and thus could be also used on cardiac CT acquisitions with dual-energy [30, 31].

The following study limitations must be acknowledged. First, there were inherent drawbacks of the retrospective study design. Second, the study was conducted with only one CT scanner and with a relatively small number of patients, limiting the generalizability of the technique. This hold particularly true for the cut-off value indicating acute MI, which might differ in other patient populations and when using other CT scanners. Third, we performed two-dimensional TA on selected ROIs rather than using a three-dimensional analysis of the entire LV myocardium. Also, TA was restricted to the slice showing the largest area of perfusion impairment, and it remains to be elucidated whether TA performs equally well if infarcted areas are smaller. Fourth, we used as a pathological model patients with acute MI where the necrotic area in MI usually is well demarcated and can be detected also visually, not requiring advanced quantitative texture features. However, this study was intended to demonstrate the general feasibility of TA in cardiac CT. Fifth, we did not investigate the influence of other protocol parameters such as reconstruction filters, scanner type and data acquisition technique, and other issues such as reconstruction phases and heart rate of the patients on TA features. Finally, we only computed a limited set of texture features instead of analyzing several hundred features, however, those have been repeatedly proven useful in the analysis of medical images after normalization [32–36].

In conclusion, our first experience study indicates that TA based on cardiac CT image data is feasible and allows for defining objective and quantitative metrics enabling the distinction between healthy subjects and those with acute MI. Future studies must address the clinical value of the TA technique in more subtle myocardial disease and whether TA features improve the diagnostic and prognostic performance as compared to a visual analysis of CT images alone.

## Supporting information

S1 Dataset Texture analysis features of controls and patients with acute MI (Reader 1). (XLSX)

S2 Dataset Texture analysis features of controls and patients with acute MI (Reader 1, second time). (XLSX)

S3 Dataset Texture analysis features of controls and patients with acute MI (Reader 2). (XLSX)
